# Habitat specificity of a threatened and endemic, cliff-dwelling halophyte

**DOI:** 10.1093/aobpla/plu032

**Published:** 2014-06-18

**Authors:** Ana D. Caperta, M. Dalila Espírito-Santo, Vasco Silva, Ana Ferreira, Ana P. Paes, Ana S. Róis, José C. Costa, Pedro Arsénio

**Affiliations:** 1 Plant Diversity and Conservation Group, Centro de Botânica Aplicada à Agricultura, Instituto Superior de Agronomia, University of Lisbon, Tapada da Ajuda, 1349-017 Lisbon, Portugal; 2 Centro de Ecologia Aplicada ‘Prof. Baeta Neves’, Instituto Superior de Agronomia, University of Lisbon, Tapada da Ajuda, 1349-017 Lisbon, Portugal; 3 Cascais Municipal Environment Company, Complexo Multiserviços, Estrada de Manique no. 1830, Alcoitão, 2645-138 Alcabideche, Portugal

**Keywords:** Agamospermic species, cliff-dwelling species, conservation, habitat specificity, halophyte, *Limonium*.

## Abstract

Coastal areas and other saline environments are major contributors to regional and global biodiversity patterns. In these environments, rapidly changing gradients require highly specialized plants like halophytes. In European coastal cliff-tops, rocky and sandy seashores, and saltmarshes, typical halophytes from the genus *Limonium* are commonly found. Among them, the aneuploid tetraploid (2*n* = 4*x* = 35, 36, 37) *Limonium multiflorum*, endemic to the west coast of Portugal, is an interesting case study for investigating the ecology and conservation of a halophyte agamospermic species. Although it is listed in the IUCN red list of threatened species, information on its population size or rarity, as well as its ecology, in some respects is still unknown. Field surveys in the largest known population were performed (Raso cape, Portugal) in order to determine habitat requirements and conservation status. A total of 88 quadrats were monitored, 43 of which contained at least one *L. multiflorum* individual. For each sampled quadrat, four abiotic and four biotic variables as well as two spatially derived variables were recorded. Principal component analysis and cluster analysis showed narrow habitat specificity for this species which appeared to be intolerant to competition with invasive alien plants. We conclude that *in situ* conservation in a local ‘hotspot’ of this rare and vulnerable species emerges as a priority in order to ensure that biodiversity is not lost.

## Abstract (precis)

Coastal cliff-tops are specific saline environments, where only highly specialized halophytes can thrive. Limonium spp. are commonly found in these ecological conditions, many of them being considered as threatened or with an unknown conservation status. The habitat requirements of Limonium multiflorum, an apomictic halophyte endemic to western Portugal, were investigated. Results showed the species narrow habitat specificity as well as its intolerance to competition with invasive alien plants. We conclude that in situ conservation of this rare and vulnerable species emerges as a priority in order to ensure that its biodiversity is not lost.

## Introduction

Habitat assessment is a fundamental requirement for species conservation. Attempts to set plant conservation priorities revealed the need to consider attributes such as species ecological specificity, geographical rarity and rate of threat (Domínguez Lozano *et al*. 2003; Pärtel *et al*. 2005). Furthermore, a study on plant life history traits in rare versus common *taxa* also showed that infrequent species exhibited a narrower geographical range and more habitat specialization than their common relatives (Farnsworth 2007). Thus, knowledge on habitat requirements of a rare species is crucial, in particular in the case of agamospermic species (defined by an asexual breeding system) (Domínguez Lozano *et al*. 2003), to help establish the best conservation methods.

In Europe, coastal vegetation presents lower diversity values when compared with other world regions (Mucina 2013) but European dry coastal terrestrial habitats, which include maritime rocks, sea-cliffs and coastal slopes, show higher values in terms of plant diversity (van der Maarel 1993; van der Maarel and van der Maarel-Versluys 1996). Among the best represented flowering plant families of these habitats are the *Plumbaginaceae* with nearly 90 % of coastal species, including most members from the genus *Limonium* (Kubitzki 1993; van der Maarel and van der Maarel-Versluys 1996). This cosmopolitan halophytic genus comprises annual and perennial species found on cliff-tops, rocky and sandy seashores, and saltmarshes (Erben 1993; Kubitzki 1993). These habitats are an important source of endemics (van der Maarel and van der Maarel-Versluys 1996), and nearly 37 % of all typical littoral species are considered threatened either because they have an extremely local distribution or because they are in decline as a result of negative human impacts in coastal areas (van der Maarel and van der Maarel-Versluys 1996).

Knowledge of habitat requirements of threatened populations from rare species selected for conservation is crucial for assuring their viability (Simberloff 1988; Brussard 1991; Schemske *et al*. 1994; Heywood and Iriondo 2003), since the establishment and expansion of a species is dependent on growth under favourable ecological conditions (e.g. Baumberger *et al*. 2012). Increasing a species' survival prospects through reintroduction or reinforcement (increase population size and diversity) of native species (Akeroyd and Wyse Jackson 1995), and knowing ecological processes in combination with demographic and genetic processes and breeding systems is therefore essential (Godefroid *et al*. 2011). However, objective data for documenting habitat preferences of rare plant species are relatively scarce, in particular those on ocean-exposed high cliff-tops subjected to salt spray transported inland by wind (Salisbury 1952; Maun 2004; Frederiksen *et al*. 2006).

The Portuguese coast is known for the richness of its flora due to a singular biogeographic position (Braun-Blanquet *et al*. 1972; Asensi *et al*. 1993; van der Maarel and van der Maarel-Versluys 1996). About 35 % of all Portuguese Natura 2000 habitats consist of coastal habitats, including Atlantic ocean-exposed cliff-tops (Costa *et al*. 2007; Martins *et al*. 2013). These locations present the original flora and vegetation on limestone, sandstone and marly clay areas, and include the rare and endemic cliff-dwelling *Limonium multiflorum* (Erben 1978, 1993; Costa *et al*. 2000; Espírito-Santo *et al*. 2012). This species is listed in Annex II of Habitats Directive (Council Directive 92/43/EEC 1992) and in the IUCN red list of threatened species (IUCN 2013), and most of its known populations are located in Portuguese NATURA 2000 Sites of Community Importance (EC 2013). Although *L. multiflorum* is considered a Portuguese crop wild relative (Magos-Brehm *et al*. 2008) and has an assessed conservation status of ‘Least Concern’ in the IUCN red list (IUCN 2013), information on its ecological preferences, essential for recovery plans, especially population restoration, population augmentation or population reintroduction is lacking.

In the present study we assessed habitat requirements for *L. multiflorum*. Two main questions were addressed: (i) What are the main abiotic and biotic variables favourable for its persistence? (ii) What is the vegetation cover and respective species composition associated with its presence?

## Methods

### Study species

**Figure 1.**
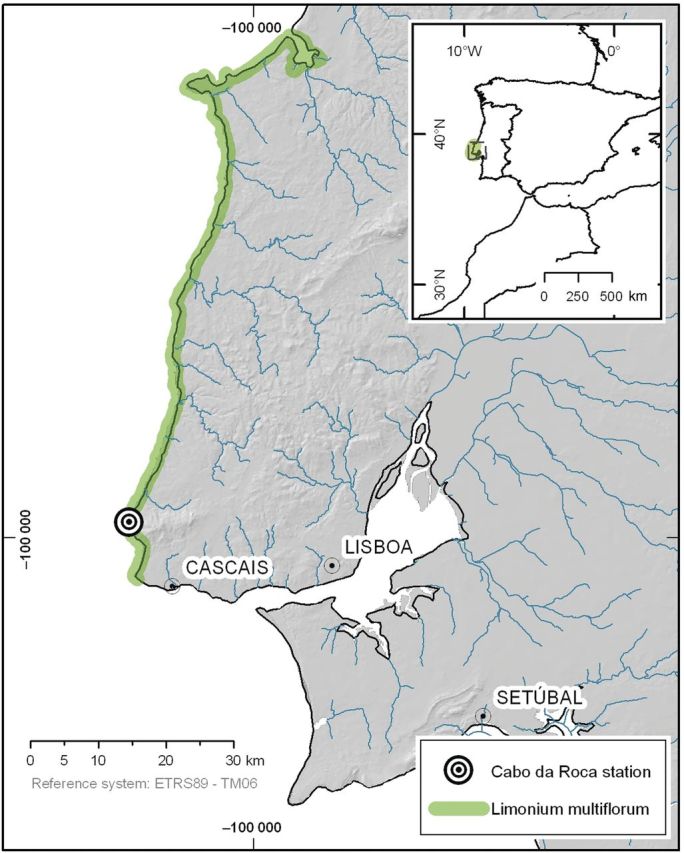
Map of *L. multiflorum* distribution in the western coast of Portugal.

*Limonium multiflorum* (2*n* = 4*x* = 35, 36, 37) (Erben 1993; Róis *et al*. 2012) is a perennial species endemic to a 120-km-long shoreline stretch in western Portugal (ICNF 2013) (Fig. 1) (Espírito-Santo *et al*. 2012). It is mainly found in NATURA 2000 habitat 1240 *Vegetated sea cliffs of the Mediterranean coasts with endemic Limonium* spp. and habitat 1330 *Atlantic salt meadows* (APA 2011). Along the western coast, populations are found on different cliffs separated by unsuitable habitats such as acid rock cliffs or steep slopes (mostly granite and syenite), sandy beaches or pinewoods. It occurs in small populations from <10 individuals to 1000 flowering plants on average (Caperta *et al*. 2013; Róis *et al*. 2013). Seedling emergence is mainly observed in autumn, following dispersal of seeds in summer. However, a persistent seed bank is not formed in such a thin soil and only a small amount of seeds originate seedlings (A.D.C. and A.S.R., unpubl. res.). Plants then grow as vegetative rosettes for several years on cliff-tops and in nearby rocky areas with a high degree of exposure to salt spray and salt-laden winds from the Atlantic Ocean (Caperta *et al*. 2013). Flowering mainly occurs in spring and summer (April to July), although in some years, it is also observed in November and February (A.D.C. and A.S.R., unpubl. res.).

### Study site

**Figure 2.**
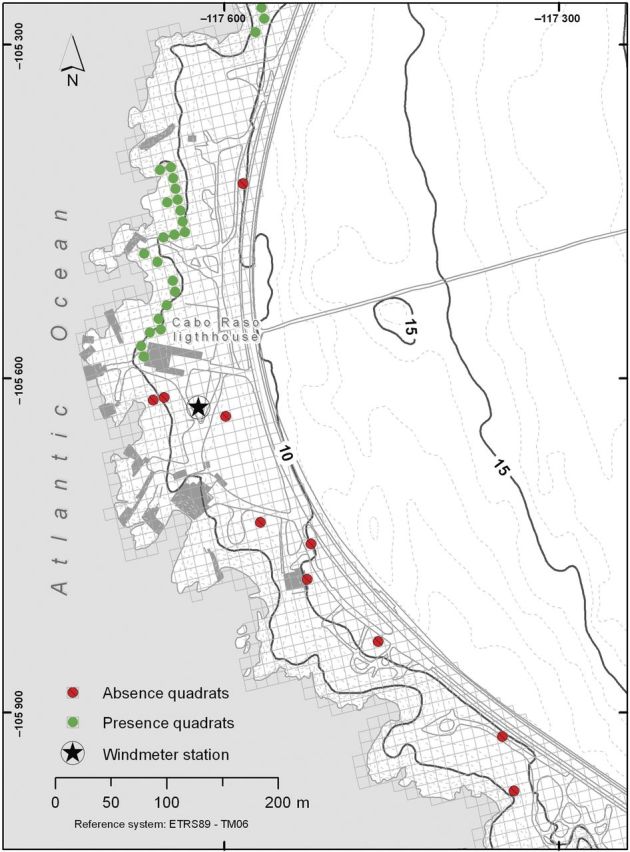
Schematic drawing of the quadrat sampling of *L. multiflorum* in the coast of Raso cape.

**Figure 3.**
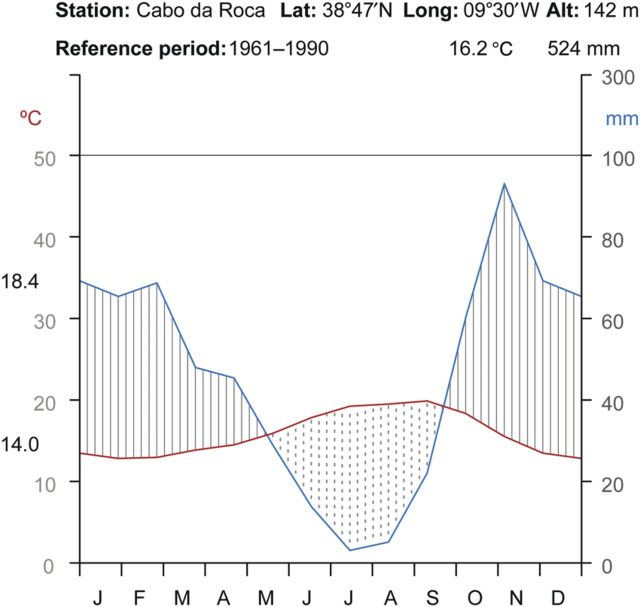
Thermopluviometric diagram based on the weather station in the coast of Raso cape. The red line corresponds to mean monthly temperature (°C) and the blue line corresponds to mean monthly precipitation (mm).

**Figure 4.**
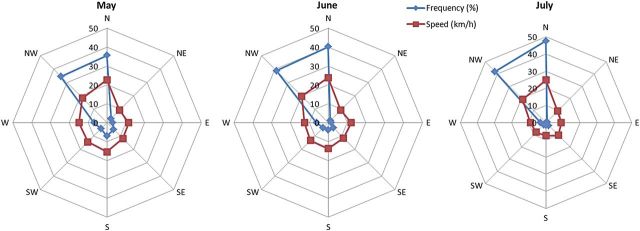
Wind regime diagrams based on the weather station in the coast of Raso cape during *L. multiflorum* flowering peak (May–July).

Our study was conducted in Raso cape, a broad promontory found in the west of Lisbon (municipality of Cascais, district of Lisbon, Portugal; mean location coordinates are 38°42′34″N and 9°29′12″W) (Fig. 2). Presently, the largest known population of *L. multiflorum* (about 1000 individuals) is found at this site, within an area of ∼0.6 km^2^, inside a Site of Community Importance (SCI Sintra/Cascais PTCON0008) for the Mediterranean biogeographical region. This cape consists of gently folded limestone, forming a rocky shoreline with low cliffs and numerous deep incisions along fault lines or abraded mylonitic rocks (Scheffers and Kelletat 2005). In terms of biogeographical typology this site is included in the Olissiponean District, Dividing Portuguese Sector, Sadensean-Dividing Portuguese Subprovince and Coastal Lusitan-Andalusian Province (Costa *et al*. 1998*a*; Rivas-Martínez 2007). The region's climate is well represented by the thermopluviometric diagram of Cabo da Roca station (Fig. 3), which presents a typical hyperoceanic Mediterranean climate pattern, as indicated by the low variation of average monthly temperatures and the existence of more than two consecutive dry months in the summer period. Another noteworthy feature of the region's climate is the high frequency of relatively strong winds coming from the northern and northwestern sectors (Fig. 4). Bioclimatically speaking (*sensu* Rivas-Martínez 2007), most of this region falls in the upper thermomediterranean thermotype and in the lower sub-humid to upper dry ombrotypes (Mesquita and Sousa 2009; Monteiro-Henriques 2010). Here, the predominant plant community is *Limonietum multiflori-virgati* (Costa *et al*. 1998*b*, 2012) which integrates *L. multiflorum*, *L. virgatum*, *Dactylis marina*, *Plantago coronopus* and *Crithmum maritimum*, among other species. This area encompasses different human occupations, namely buildings and other built structures like seafood tanks, off-road motorized driving and tourism which contribute to population fragmentation.

#### Sampling design

Field surveys used a grid of 10 × 10 m quadrats which was overlaid over the aerial photographs of the site and later transferred to the site using global positioning system (GPS) receivers. A total of 88 quadrats were surveyed, 43 of which contained at least one *L. multiflorum* individual (presence quadrat) and the remaining 45 were randomly selected quadrats containing no individuals from this species (absence quadrat) (Fig. 2). The random selection procedure was performed using R software's ‘sample’ routine (R Core Team 2013). In quadrats where *L. multiflorum* was present censuses were carried out from April to June 2013. For each sampled quadrat four abiotic variables, namely rock formation (RockForm), cobble (Cobble), coarse sand (CoarseSd) and fine sand (FineSand) (all expressed in %), were recorded. Four biotic variables related to vegetation cover (Coverage) as well as invasive non-native species (INNS) cover, dead organic matter (lignified or not) (DOM) cover and litter (Residues) cover were also estimated (expressed in %) in each quadrat. To facilitate data collection, each quadrat was then sub-divided into sub-quadrats of 1 m^2^, and the percent coverage value obtained for the sub-quadrats of the same 100 m^2^ quadrat were averaged. Two spatial variables, distance from coastline (Dist_coa) and mean quadrat slope (Mean_slo), were also derived using the ArcGIS^®^ 10.0 software, by ESRI.

### Data analysis

To define *L. multiflorum* habitat requirements we performed a principal component analysis (PCA) on the resulting data matrix using abiotic, biotic and spatially derived variables. In order to discriminate the vegetation structure and composition in the monitored quadrats, a cluster analysis using Euclidean distance and Ward clustering method (Ward 1963) was performed using the set of quadrats containing *L. multiflorum*. To ensure that the group of quadrats (clusters) had the same influence from the environmental variables, Kruskal–Wallis tests of variance were performed (Sokal and Rohlf 1997; Zar 2010). To complete habitat characterization of *L. multiflorum,* the most frequent and abundant plant species within each cluster was defined as described in Baumberger *et al*. (2012). The mean coverage of each species within each group was also calculated. All statistical analyses were conducted using the STATISTICA software (StatSoft V10). Bioclimatic and biogeographic nomenclature followed the proposals of Rivas-Martínez (2007). Taxonomic nomenclature followed Menezes de Sequeira *et al*. (2011).

## Results

### Influence of environmental variables on the presence/absence of *L. multiflorum*

On the western coast most *L. multiflorum* populations occur at inaccessible cliff sites and/or show very small size (Róis *et al*. 2013). Under these circumstances, this study was conducted at the best accessible site and on the largest known population for this species, in Raso cape. Study site rocks were bare of vegetation up to ∼7–8 m asl, due to strong exposure to Atlantic storms and waves over the years.

**Figure 5.**
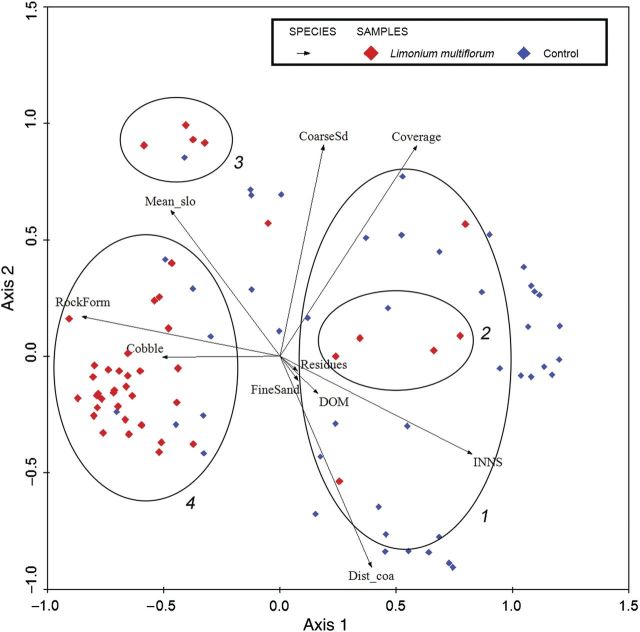
Two first axes of PCA based on *L. multiflorum* presence and absence quadrats. Red symbols mean that *L. multiflorum* was recorded whereas blue symbols signify that it was absent. Axis 1 represents 44.8 % of variation, and Axis 2, 19.7 %. CoarseSd, coarse sand; Cobble, cobble; Coverage, vegetation cover; Dist_coa, distance from coast; DOM, dead organic matter cover; FineSand, fine sand; INNS, invasive non-native species cover; Mean_slo, mean slope; Residues, litter cover; RockForm, rock formation.

Of the 88 quadrats sampled, only 43 contained at least one *L. multiflorum* individual (presence) while control quadrats did not show any individual from this species (absence). Considering the presence quadrats, *L. multiflorum* mean coverage was very low (about 0.67 %). Only one of the variables measured in each quadrat fitted a normal distribution (variable ‘Mean_slo’), and the other nine failed to do so, even after a logarithmic transformation. Therefore, the remaining analyses were performed using the original (untransformed) values. Remarkably, visual inspection of PCA revealed that in the first two represented ordinations, quadrats showing the presence of *L. multiflorum* individuals were highly correlated with rock formation (variable ‘RockForm’) and non-correlated with high coverage (variable ‘vegetation cover’) (Fig. 5). Considering the first two principal components of PCA, the percentage of accumulated variance was 64.4 % (Fig. 5). The first axis accounted for 44.8 % of the variance and was explained by vegetation cover, while the second axis accounted for 19.7 % of the variance explained by distance from coast (variable ‘Dist_coa’).

### Influence of environmental variables in *L. multiflorum* persistence

**Figure 6.**
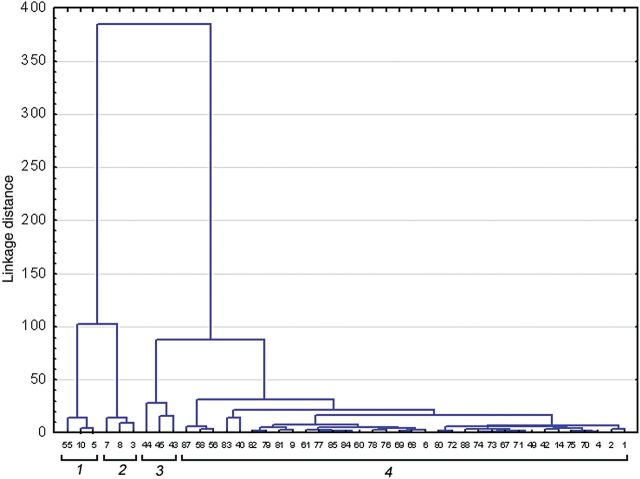
Cluster dendogram of the four clusters containing *L. multiflorum*. The dissimilarity between groups was calculated based on species variables using Euclidean distance and the Ward aggregation method. Cluster definition 1 accounts for a high percentage of *C. edulis* (>65 %); Cluster 2 by a moderate percentage of *C. edulis* (<35 %); Cluster 3 is defined by *A. welwitschii* (9.67 %) and *P. incurva* (21.33 %); and Cluster 4 represents quadrats with the highest *L. multiflorum* frequency (0.84 %).

A cluster analysis was performed using the dataset of quadrats showing *L. multiflorum* presence. The data obtained revealed the existence of four floristic groups among the sampled quadrats (Fig. 6; Tables 1 and 2). Environmental conditions differed between Group 4 and the other three floristically defined groups. Clusters 3 and 4 were clearly individualized in PCA. The first groups (Groups 1, 2 and 3) presented high vegetation coverage percentage (variable ‘coverage’) combined with low rock outcrop percentages (variable ‘RockForm’) whereas the opposite occurred in Group 4, which showed the highest *L. multiflorum* cover percentage. Also, in this latter group low percentages of invasive non-native species (variable ‘INNS’) were found. Although in both Groups 1 and 2, ‘INNS’ percentages were greater than those in the other groups, Group 2 differed in terms of fine sand percentages (variable ‘FineSand’).

**Table 1.** Mean values ± standard error of abiotic, biotic and spatial-related variables in each of four environmental groups based on the presence data of PCA. Bold letters within a row indicate non-significant with post hoc multiple comparison Tukey's test (*α* = 0.05; *P* < 0.001). *P* value <0.001. ns, non-significant; CoarseSD, coarse sand; Cobble, cobble; Coverage, vegetation cover; Dist_coa, distance from coast; DOM, dead organic matter cover; FineSd, fine sand; INNS, invasive non-native species cover; Mean_slo, mean slope; Residues, litter cover; RockForm, rock formation.

	Environmental groups			
	1 (*n* = 3)	2 (*n* = 3)	3 (*n* = 3)	4 (*n* = 34)
Coverage (%)*	54.83 ± 47.54 (**a**)	47.17 ± 11.07 (**a**)	37.67 ± 11.68 (**a**)	3.10 ± 7.44 (**b**)
FineSd (%)*	0.00 ± 0.00 (**a**)	3.17 ± 2.75 (**b**)	0.00 ± 0.00 (**a**)	0.00 ± 0.00 (**a**)
INNS (%)*	73.00 ± 6.24 (**a**)	33.00 ± 3.46 (**b**)	0.00 ± 0.00 (**c**)	0.88 ± 2.55 (**c**)
RockForm (%)*	5.00 ± 4.00 (**a**)	6.00 ± 5.29 (**a**)	40.00 ± 11.79 (**a,b**)	74.49 ± 23.58 (**b**)
CoarseSd (%) ns	5.67 ± 8.14	0.00 ± 0.00	11.67 ± 4.16	3.37 ± 9.17
Dist_coa (m) ns	11.36 ± 8.24	16.51 ± 3.95	0.00 ± 0.00	16.31 ± 9.88
Cobble (%) ns	4.77 ± 6.60	45.00 ± 8.23	8.67 ± 10.017	13.63 ± 19.85
DOM (%) ns	2.67 ± 4.62	0.00 ± 0.00	5.33 ± 9.24	0.41 ± 1.23
Mean_slo (m) ns	16.63 ± 5.66	17.25 ± 0.82	68.92 ± 28.97	25.21 ± 21.50
Residues (%) ns	0.33 ± 0.58	0.42 ± 0.52	0.00 ± 0.00	0.09 ± 0.19

**Table 2.** Mean coverage (%) of the most frequent species in each of the four environmental groups (clusters) based on the presence data of PCA.

Floristic list		Mean coverage (%)			
		1	2	3	4
Psammophilous	*Armeria welwitschii* Boiss*.*	1.17	2.34	9.67	0.03
	*Inula crithmoides* L.	0.00	0.17	0.12	1.33
	*Herniaria maritima* Link	0.00	0.67	0.00	0.07
	*Lobularia maritima* (L.) Desv. subsp. *maritima*	0.00	0.00	0.20	0.00
	*Parapholis incurva* (L.) C.E.Hubb.	0.50	0.00	21.33	0.70
	*Helichrysum italicum* (Roth) G.Don subsp. *picardi* (Boiss. & Reut.) Franco	0.33	0.00	0.17	0.03
	*Andryala arenaria* (DC.) Boiss. & Reut. subsp. *arenaria*	0.17	0.33	0.58	0.06
	*Otanthus maritimus* (L.) Hoffmanns. & Link	0.00	2.75	0.00	0.02
	*Elymus farctus* (Viv.) Runemark ex Melderis subsp. *boreo-atlanticus* (Simonet & Guin.) Melderis	3.33	0.77	1.00	0.98
	*Lotus creticus* L.	0.00	0.01	0.25	0.40
	*Medicago minima* (L.) L.	0.00	0.50	1.00	0.03
	*Crucianella maritima* L.	0.13	0.00	0.00	0.03
	*Cakile maritima* Scop.	0.00	0.00	0.00	0.01
	*Dactylis smithii* Link subsp. *marina* (Borrill) Parker	0.02	0.01	0.25	0.09
Chamosphilous	*Limonium ovalifolium* (Poir.) Kuntze	0.00	0.00	1.33	0.03
	*Limonium virgatum* (Willd.) Fourr.	0.17	0.00	0.07	0.07
	*Plantago coronopus* L.	0.02	0.17	0.75	0.07
	*Limonium multiflorum* Erben	0.03	0.05	0.11	0.84
	*Frankenia laevis* L.	0.83	1.67	1.00	1.03
	*Crithmum maritimum* L.	0.30	3.67	0.23	0.66
	*Catapodium marinum* (L.) C.E.Hubb.	0.00	0.00	0.00	0.03
Nitrophilous	*Carpobrotus edulis* (L.) N.E.Br.	73.00	33.00	0.00	0.88
	*Beta maritima* L.	0.08	0.33	0.50	0.28
	*Daucus halophilus* Brot.	0.00	0.00	0.00	0.01
	*Leontodon taraxacoides* (Vill.) Mérat subsp.*taraxacoides*	0.00	0.00	0.00	0.01
	*Parapholis filiformis* (Roth) C.E.Hubb.	0.50	0.00	0.00	0.06
	*Polypogon maritimus* Willd.	0.00	0.00	0.00	0.02

Vegetation composition also varied between clusters. Cluster 1 was dominated by *Carpobrotus edulis* with a coverage percentage >65 % whereas Cluster 2 presented a moderate coverage of *C. edulis* (<35 %). In this cluster significant mean coverage was observed for psammophilous species like the perennials *Armeria welwitschii* and *Elymus farctus* subsp. *boreo-atlanticus*. As for Cluster 3, significant frequencies of the perennial *Limonium ovalifolium* and the therophyte *Parapholis incurva* were revealed. Finally, Cluster 4 which had the greatest coverage percentage of *L. multiflorum* (0.835 %) of all clusters appeared to be associated with chasmophytic species such as *C. maritimum*, and other perennials like *Frankenia laevis* and *Inula crithmoides*.

## Discussion

### *L. multiflorum* displays narrow habitat specificity

In this study we show that *L. multiflorum* grows on sea-cliffs preferentially up to 50 m asl in karstic crevices within exposed rocks more or less filled with substrate, or on shallow soil above the rock strata and on scree slopes. The presence of *L. multiflorum* was favoured by the presence of rock formation, cobble, low percentage of vegetation coverage, low frequency of invasive non-native species and closest distance to coast. In fact, as pointed out by Warming (1909), *L. multiflorum*'s preference for salt-rich rock crevices seems to be a strategy to use such locations as refuges, thus avoiding competition with many other coastal plants that are unable to colonize such habitats. Furthermore, as it happens in endemic *Limonium* spp., populations tend to be fragmented and usually show low population sizes as a consequence of many social and economic activities like urbanization, tourism and traffic (Rodríguez *et al*. 2003; Palop-Esteban *et al*. 2007; Suárez-García *et al*. 2009; Khan *et al*. 2012; Róis *et al*. 2013) on their habitats. This is also the case of *L. multiflorum* (ICNF 2013), in particular, in the Raso cape population (A.F. and V.S., unpubl. res.).

Vegetation composition significantly differs among the four floristically defined groups. Cluster 1 is dominated by the alien plant *C. edulis* with *E. farctus* subsp. *boreo-atlanticus*'s due to nitrophilous coarse sand dunes corresponding to *Elytrigietum junceo-boreoatlanticae* association (Costa *et al*. 2012). The presence of *L. multiflorum* in this cluster can be explained by 5 % of rock outcrop, uncovered with sand. Cluster 2 includes the farthest quadrats from the sea where rock depressions filled up with fine sand and cobble appear. In this cluster *A. welwitschii*, *F. laevis* and *L. multiflorum* predominate. This cluster presents a poor floristic composition, as it happens in the previous cluster, due to invasion of *C. edulis*, possibly related with the proximity to roads (higher nitrification)*.* Cluster 3 is typically formed by species of rocky substrate, with a high slope and direct influence of wind loaded with salt. Here *L. ovalifolium*, *C. maritimum*, *A. welwitschii*, *F. laevis*, *L. multiflorum*, *P. coronopus* form the perennial community *Limonietum multifloro-virgati* (Costa *et al*. 1998*b*, 2012). In their clearings *P. incurva*, *Medicago minima*, *Andryala arenaria* form the poor, annual association *Parapholido incurvae–Catapodietum marini*. Also, the presence of *Lotus creticus* is due to coarse sand. Cluster 4 also exhibits species that constitute the *Limonietum multifloro-virgati* association (Costa *et al*. 1998*b*, 2012), namely *C. maritimum*, *L. multiflorum*, *I. crithmoides*, *F. laevis*, *P. coronopus*. The referred species are at their ecological optimum in this rocky sea-cliff cluster although a small percentage of coarse sand explains the presence of *E. farctus* subsp. *boreo-atlanticus* and *L. creticus.* Plant survival in this ecological context is difficult due to low availability of fresh water, low level of essential nutrients and the abrasive action of strong winds laden with salt and increasing conditions of extreme dryness (Costa *et al*. 1998*b*). To overcome this some plants respond with adaptations, such as adopting the hemicryptophyte or therophyte life forms, presenting succulent leaves and/or salt glands in the leaves (Warming 1909; Costa 2001; Grigore *et al*. 2014).

### *L. multiflorum* appears to be intolerant to competition with invasive species

Our results show that both native and non-native vegetation cover are negatively correlated with *L. multiflorum* presence, emphasizing its preferences for sites where competition with other species is low. Among the species having a greater negative effect in their persistence is the exotic, invasive, crawling *C. edulis*. This species commonly invades coastal habitats in Mediterranean Europe forming mantles on maritime rocks, cliffs and sand dunes (D'Antonio 1990; Traveset *et al*. 2008; Pyšek *et al*. 2013) competing directly with native plant species, suppressing the growth and establishment of other plants (D'Antonio and Mahall 1991; Vilà *et al*. 2006), altering certain soil parameters (Vilà *et al*. 2006; Conser and Connor 2009) or creating litter accumulation on the soil surface (Molinari *et al*. 2007). Although *C. edulis* seems to develop well on rocky substrates at sites where seagulls' (*Larus* sp.) nutrient-rich droppings are frequent (M.D.E.-S., unpubl. res.), in our study it seems to compete mainly with species showing preference for sand substrates. Remarkably, some littoral species show strong resilience to *C. edulis* invasion, like the narrow endemic cliff-species *Armeria pseudoarmeria* (Pinto-Ricardo *et al*. 2010) or other *Limonium* spp. (Snogerup 1971; Suehs *et al*. 2003; Campos *et al*. 2004).

### Conservation issues

For the effective protection of the rare and endemic *L. multiflorum* not only ecological data but also life story, demography and genetic data should be taken into account to ensure that maximum genetic variation is preserved. In this context, one of the factors to be considered in conserving *L. multiflorum* is related with chromosome polymorphisms since euploid and aneuploid cytotypes occur within populations (Róis *et al*. 2012). Although, it was first described as an unbalanced aneuploid tetraploid (2*n* = 4*x* = 35) (Erben 1978), both unbalanced and balanced tetraploid cytotypes (2*n* = 4*x* = 35, 36, 37) and diverse karyological polymorphisms were found within and among populations, with greater variability in the Raso cape population (Róis *et al*. 2012). This has also been observed in other *Limonium* taxa (e.g. Castro and Rosseló 2007), and even in a variety of genera from different plant families (e.g. *Brassicaceae*, Böcher 1954; *Campanulaceae*, James 1965; *Onagraceae*, Bloom 1974; *Plumbaginaceae*, Erben 1978; *Poaceae*, Sieber and Murray 1981; *Ranunculaceae*, Borchers-Kolb 1983). These cytological differences should be considered to ensure that maximum genetic variation is preserved. Furthermore, the resultant combination of incompatible cytotypes could result in the failure of reintroduced plants to reproduce and may bring reproductive instability to augmented populations (Young and Murray 2000; Ennos *et al*. 2005; Severns and Liston 2008).

Noticeably, male sterility and gynodioecism are found in plants from experimental collections (Róis *et al*. 2012) and field plants (Heike Sprenger and A.D.C., unpubl. res.). However, these plants are capable of producing large numbers of viable seeds with variable ploidy levels, as revealed by flow cytometry seed screening (Róis *et al*. 2012) and reproduce through apomixis (A.S.R. and A.D.C., unpubl. res.). This form of uniparental reproduction is found in ‘taxonomically complex groups’ like in other *Limonium* spp. (Erben 1978; Palacios *et al*. 2000; Lledó *et al*. 2005) and also in dandelions (*Taraxacum* spp.; van Dijk 2003), blackberries (*Rubus fruticosus* agg.; Salvini *et al*. 2006) and in *Ranunculus* spp. (Hörandl *et al*. 2009), which generates a diverse mixture of related individuals (Ennos *et al*. 2005).

While some *L. multiflorum* populations are located in natural parks (Parque Natural Sintra-Cascais) and other areas under legal protection (e.g. Site of Community Importance—SCI Sintra/Cascais PTCON0008; SCI Peniche/Sta Cruz PTCON0056), in the past decades no information on population size along its distribution range has been available. However, we hypothesized that population decline might have occurred due to increasing urban development in the coastal areas and the impact of tourism. For instance, in Raso cape, sightseeing tourists, sports fishing and other socio-economic activities, together with competition associated with invasion by non-native species are cumulatively leading to population decline (V.S., A.F. and A.D.C., unpubl. res.). Notably, methylation-sensitive amplified polymorphism (MSAP) data from *L. multiflorum* natural populations reveal low levels of genetic/epigenetic diversity (Róis *et al*. 2013). Altogether these data mean that none of the populations analysed could restore the genotypic diversity observed in any other *L. multiflorum* population. Hence, habitat protection emerges as the top priority to prevent population extinction of the narrow-specialist *L. multiflorum*. For the conservation of this species, management actions such as those referred by Laguna *et al*. (2013), namely limiting access to populations, for example, the experimental exclusion of herbivores by means of fences to protect populations of *L. dufourii* in ‘Marsh dels Moros’ (Spain) and to perform selective removal and control of invasive species (Laguna *et al*. 2013), are required. Other technical solutions could be population reinforcements, reintroduction or establishment of new populations, the goal of which is the introduction of new specimens. In the case of *L. multiflorum*, attempts to create new populations through experimental actions like sowing seeds directly *in situ* did not prove to be successful (A.D.C. and V.S., unpubl. res.) but population reinforcements by planting newly produced specimens using seed stock that originated from the same natural populations (e.g. Raso cape) appears to give better results (Caperta *et al*. 2013). Complementary *ex situ* conservation actions for this species such as collection and storage of seeds preserved in João do Amaral Franco Seed Bank (Ajuda Botanical Garden) have also been conducted (Caperta *et al*. 2013) with the hope that the genetic diversity contained in these collections could be representative of natural populations. Thus, to conserve *L. multiflorum*, it is preferable to develop small, localized experimental actions as Laguna *et al*. (2013) pointed out. In fact, only moderate success is reported for reintroduction projects due, for example, to insufficient long-term monitoring following reintroduction and/or lack of understanding of the underlying reasons for decline in existing plant populations (e.g. Godefroid *et al*. 2011).

## Conclusions

According to Rabinowitz' scheme (1981), in which species were classified into categories according to their geographic range, habitat specificity and local population size, *L. multiflorum* can be classified as a ‘classic rarity’ since it presents both narrow geographic range and narrow habitat specificity, thus being considered a restricted endemic. Furthermore, species' populations observed throughout its entire range consistently present small sizes and low levels of genetic diversity as revealed by population genetic and epigenetic studies using MSAP markers (Róis *et al*. 2013). Taking the above into consideration, as well as diverse social-economic impacts exerted on its populations, we considered that *L. multiflorum* deserves a status of ‘Vulnerable’ in the IUCN red list.

As already pointed out for other ‘taxonomically complex groups’ (Ennos *et al*. 2005), conservation of rare and endemic *L. multiflorum* is best achieved by facilitating evolutionary interactions among its members that generate and maintain their taxonomic biodiversity.

## Sources of Funding

Our work was funded by Fundação para a Ciência e Tecnologia (FCT) (Portugal) (PEST-OE/AGR/UI0240/2011). A.D.C. (Researcher, CBAA/ISA) and A.S.R. (SFRH/BD/62542/2009 grant) were supported by FCT.

## Contributions by the Authors

A.D.C. and P.A. designed and coordinated the study. M.D.E.-S. coordinated the fieldwork and A.D.C., M.D.E.-S., A.P.P. and A.S.R. performed the fieldwork. A.F. and V.S. processed the raw data and carried out statistical analysis. J.C.C. and M.D.E.-S. performed the phytosociological analysis. A.D.C. and P.A. drafted the manuscript. All authors read and approved the manuscript.

## Conflicts of Interest Statement

None declared.
